# Artificial Intelligence for Tooth Detection in Cleft Lip and Palate Patients

**DOI:** 10.3390/diagnostics14242849

**Published:** 2024-12-18

**Authors:** Can Arslan, Nesli Ozum Yucel, Kaan Kahya, Ezgi Sunal Akturk, Derya Germec Cakan

**Affiliations:** 1 Department of Orthodontics, Faculty of Dentistry, Yeditepe University, Istanbul 34728, Turkey; 2 Department of Orthodontics, Hamidiye Faculty of Dental Medicine, University of Health Sciences, Istanbul 34668, Turkey

**Keywords:** artificial intelligence, cleft lip and palate, tooth detection, tooth numbering

## Abstract

**Introduction:** Cleft lip and palate patients often present with unique anatomical challenges, making dental anomaly detection and numbering particularly complex. The accurate identification of teeth in these patients is crucial for effective treatment planning and long-term management. Artificial intelligence (AI) has emerged as a promising tool for enhancing diagnostic precision, yet its application in this specific patient population remains underexplored. **Objectives:** This study aimed to evaluate the performance of an AI-based software in detecting and numbering teeth in cleft lip and palate patients. The research focused on assessing the system’s sensitivity, precision, and specificity, while identifying potential limitations in specific anatomical regions and demographic groups. **Methods:** A total of 100 panoramic radiographs (52 males, 48 females) from patients aged 6 to 15 years were analyzed using AI software. Sensitivity, precision, and specificity were calculated, with ground truth annotations provided by four experienced orthodontists. The AI system’s performance was compared across age and gender groups, with particular attention to areas prone to misidentification. **Results:** The AI system demonstrated high overall sensitivity (0.98 ± 0.03) and precision (0.96 ± 0.04). No statistically significant differences were found between age groups (*p* > 0.05), but challenges were observed in the maxillary left region, which exhibited higher false positive and false negative rates. These findings were consistent with the prevalence of unilateral left clefts in the study population. **Conclusions:** The AI system was effective in detecting and numbering teeth in cleft lip and palate patients, but further refinement is required for improved accuracy in the cleft region, particularly on the left side. Addressing these limitations could enhance the clinical utility of AI in managing complex craniofacial cases.

## 1. Introduction

The early detection and accurate classification of dental anomalies in individuals with cleft lip and palate is crucial for effective treatment planning and improved patient outcomes. Early identification of dental abnormalities allows for timely intervention and customized treatment strategies, which can significantly enhance the overall quality of care and long-term outcomes for these patients. Individuals with cleft lip and palate often exhibit a range of dental anomalies, including missing teeth, ectopic eruption, and malformed tooth structure [1]. The presence of such anomalies complicates the identification of teeth, particularly in the cleft region, and can impede the delivery of comprehensive dental care [2]. Artificial intelligence (AI) encompasses a wide range of computational methods and technologies designed to mimic human intelligence. Among these, convolutional neural networks (CNNs) are a specific type of deep learning model that excel in image analysis tasks [3,4,5]. Advancements in AI have shown promise in automating and enhancing the identification and classification of dental features, offering a potential solution to streamline the diagnostic process [6,7,8].

In fixed prosthodontics, AI-based techniques have demonstrated the ability to accurately detect and classify dental margins, a critical step in the design and fabrication of dental restorations. Similarly, in removable prosthodontics, convolutional neural networks have been used to classify dental arches, providing valuable insights for treatment planning [9]. Moreover, AI has been applied in various other areas of dentistry, from diagnostic dentistry and patient management to orthodontics and radiology, offering improved efficiencies, precision, and patient-centric care. However, the successful integration of AI in routine dental practice faces several challenges, including limited data availability, lack of methodological rigor, and practical concerns around the value and usefulness of these solutions [10].

Nonetheless, the potential of AI-based software to enhance the identification and classification of dental anomalies in cleft lip and palate patients is promising [11]. By automating and optimizing the diagnostic process, AI-based tools could ultimately lead to more personalized, predictive, and preventive dental care, ultimately improving the quality of life for individuals with this complex craniofacial condition [12,13,14,15,16,17,18].

Panoramic radiographs are critical for the initial diagnosis of dental abnormalities in cleft lip and palate patients. However, interpreting these radiographs can be challenging, even for experienced clinicians, due to the anatomical complexity and presence of dental anomalies [15]. Several studies have explored the use of AI-based programs for tooth detection and numbering on panoramic radiographs, reporting that deep convolutional neural network systems demonstrate high sensitivity and precision in these tasks [16,17,18]. To the best of our knowledge, no studies to date have investigated the application of AI-based software for tooth detection and numbering specifically in individuals with cleft lip and palate. This study aims to evaluate the performance of an artificial-intelligence-based application in identifying and classifying teeth in patients with this craniofacial condition. The null hypothesis of this study is that the performance of the AI-based software in detecting and numbering teeth in cleft lip and palate patients does not differ significantly from expert ground truth annotations, irrespective of age or gender.

## 2. Materials and Methods

This retrospective study examined a set of panoramic radiographs from individuals in mixed dentition with cleft lip and palate, obtained from the archives of the Yeditepe University Faculty of Dentistry, Department of Orthodontics, taken from March 2019 to June 2024. A power analysis was conducted using the G*Power 3.1.9.7. (G*Power, Düsseldorf, Germany) software protocol [19]. Based on sensitivity and specificity values from a reference study [18], with a 95% confidence level (1 − α), 95% test power (1 − β), sensitivity of 0.9559, and specificity of 0.9652, the minimum sample size required for this study was calculated to be 90 participants. Therefore, a total of 100 (52 males, 48 females) individuals in mixed dentition (mean age 8.18 ± 2.24 years) with unilateral or bilateral cleft lip and palate were included in this study. To assess the impact of age-related differences in dental development, these patients were divided into three groups: 6–7 years, 8–9 years, and 10 years and older. These groups were selected due to the significant dental changes that occur during the mixed dentition period.

Panoramic radiographs that exhibited artifacts related to metal superposition, positioning errors, movement, or image distortion were excluded from the analysis. Radiographs of patients with additional severe craniofacial anomalies or those who had undergone surgical interventions affecting dental structures were also excluded. However, radiographs that displayed typical cleft-associated dental anomalies such as missing, rotated, or supernumerary teeth in the cleft region were included to ensure the study’s focus on cleft-related dental challenges. Ethical approval was obtained from the Non-Interventional Clinical Research Ethics Committee of Yeditepe University (Approval Number: 202311Y0694). The study was conducted in accordance with the principles of the Declaration of Helsinki.

**Figure 1:**
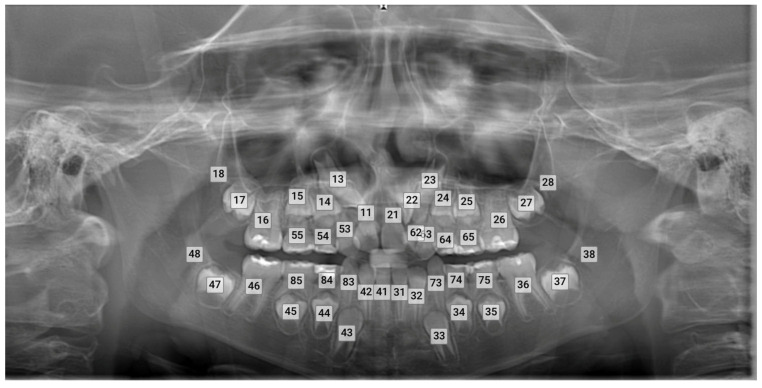
Teeth detection and numbering by the software.

All panoramic radiographs were obtained using the Morita Veraviewepocs (Morita Corp., Kyoto, Japan) and subsequently uploaded to the Diagnocat software (DC, Diagnocat LLC, San Francisco, CA, USA, https://diagnocat.com/ accessed on 24 November 2024). A radiologic report for each radiograph was generated, which served as the basis for the automatic evaluation (Figure 1). Teeth detection and numbering were performed according to the FDI notation and analyzed by five different orthodontists. In this study, ground truth annotations, manual identification, and the labeling of the correct positions and numbering of teeth, were provided by one orthodontist with 20 years of experience, two with 10 years of experience, and two with 4 years of experience. These annotations served as the reference standard for evaluating the performance of the AI-based software.

The commercially available AI system used in this study, Diagnocat, is based on deep learning methods, specifically CNNs, which are a type of deep learning architecture designed to process and analyse visual data by identifying patterns and features within images. In the context of dental imaging, these networks are trained to detect and number teeth by recognizing anatomical structures in panoramic radiographs. Diagnocat includes various specialized modules, such as region of interest (ROI) localization and tooth localization and numeration, which enhance its diagnostic capabilities. These modules are supported by state-of-the-art CNN architectures trained on extensive datasets of cone beam computed tomography (CBCT) scans and panoramic radiographs.

To assess the success of the AI model in tooth detection and numbering, the following procedures and metrics, based on two prior studies in the literature [17,18], were employed:

Initially, the true positive (TP), false positive (FP), true negative (TN), and false negative (FN) rates were calculated:

- True Positive (TP): The model correctly detects and identifies teeth.
- False Positive (FP): The model detects teeth correctly but assigns the wrong number.
- False Negative (FN): The model fails to detect or incorrectly identifies teeth.
- True Negative (TN): The model correctly identifies areas where no teeth are present.

Using these values, the following metrics were calculated:

- Sensitivity: TP/(TP + FN)
- Precision: TP/(TP + FP)
- F1 Score: 2TP/(2TP + FP + FN)
- False Discovery Rate: FP/(FP + TP)
- False Negative Rate: FN/(FN + TP)
- Error: Instances where the model incorrectly identifies a tooth in an irrelevant anatomical region where no tooth is present.

### Statistical Analysis

The data were analyzed using IBM SPSS version 23.0 software (IBM Corp., Armonk, NY, USA). The normality of the data distribution was evaluated using the Shapiro–Wilk and Kolmogorov–Smirnov tests. For comparisons between two independent groups, an independent samples *t*-test was employed for normally distributed variables, while the Mann–Whitney U test was used for non-normally distributed variables. For comparisons among three or more groups, one-way ANOVA was applied for normally distributed variables, with post hoc comparisons performed using Duncan and Tamhane tests. For non-normally distributed variables, the Kruskal–Wallis test was utilized, with post hoc comparisons conducted using the Dunn test. The results of the analyses are presented as mean ± standard deviation for normally distributed variables, and median for non-normally distributed variables. A significance level of *p* < 0.05 was considered statistically significant.

## 3. Results

A total of 100 patients with cleft lip and palate (52 males, 48 females) were included in this study. Among the male patients, 30 (57.7%) had unilateral clefts, with 21 (40.4%) on the left side and 9 (17.3%) on the right side, while 22 (42.3%) had bilateral clefts. Among the female patients, 29 (60.4%) had unilateral clefts, with 19 (39.6%) on the left side and 10 (20.8%) on the right side, and 19 (39.6%) had bilateral clefts. Table 1 summarizes the descriptive statistics for the variables studied across all patients included in the study.

### 3.1. Intraclass Correlation Coefficient (ICC)

The consistency between evaluators was assessed using the intraclass correlation coefficient (ICC). The ICC values indicated strong reliability, with intra-rater reliability ranging between 0.96 and 1, and inter-rater reliability ranging from 0.95 to 0.98. To assess intra-rater reliability, each evaluator randomly selected and re-evaluated 25 panoramic radiographs 15 days after the initial assessment. This high level of agreement confirms the accuracy and reliability of the ground truth annotations, which served as the gold standard for evaluating the AI system’s performance in tooth detection and numbering.

### 3.2. Sensitivity and Precision Across Age Groups

With the ground truth annotations established as the reference standard, the AI system’s sensitivity and precision were evaluated across the three age groups (Table 2). Sensitivity values were consistently high across all groups, with no statistically significant differences observed between the age groups (*p* = 0.840). Similarly, precision values showed no statistically significant differences among the groups (*p* = 0.172).

### 3.3. True Positive, False Positive, and False Negative Rates

Comparisons of true positive, false positive, and false negative rates between groups are shown in Table 2. The true positive rates, correctly representing detected teeth, showed significant variation between the age groups (*p* < 0.001). The older group exhibited significantly lower true positive rates compared to the younger groups, likely due to the complexity of dental structures in older patients. The false positive rate, which reflects the incorrect detection of teeth, showed no statistically significant differences across the age groups (*p* = 0.395). However, tooth number 21 had the highest false positive rate at 16%, while tooth number 62 had the highest false negative rate at 11%. This finding is particularly important, as it aligns with the fact that the majority of our patient population had unilateral left clefts, where the AI system may face more challenges in detecting teeth. Similarly, the false negative rate was consistent across age groups (*p* = 0.719).

### 3.4. F1 Score and Error Rate

The F1 score, a metric combining sensitivity and precision, was calculated for all age groups (Table 2). The F1 score remained consistently high across all groups, with no statistically significant differences observed between the age groups (*p* = 0.256). Similarly, the error rate, which reflects instances where the model falsely identifies teeth in non-dental regions, did not show significant differences between the age groups (*p* = 0.308).

### 3.5. Gender Comparisons

Gender comparisons for sensitivity, precision, and F1 score are presented in Table 3, with no statistically significant differences observed between male and female participants (*p* = 0.119, *p* = 0.441, and *p* = 0.827, respectively). However, although not statistically significant, there was a notable difference in the false negative rates between genders (*p* = 0.079), with males showing slightly higher values compared to females.

### 3.6. Age and Gender-Specific Performance

The performance of the AI system was further analyzed by dividing the participants by gender and age groups (Table 4 and Table 5). The results indicate that some significant differences were observed across different age and gender groups:

- The 6–7 years age group: Female participants showed a significantly lower false negative rate (*p* = 0.0004) and higher sensitivity (*p* = 0.0006) compared to males.
- The 8–9 years age group: Male participants exhibited higher precision (*p* = 0.0036) and a lower false discovery rate (*p* = 0.0036) than females.
- The 10+ years age group: No significant differences were observed between males and females in this age group.

**Table 4:** Comparison of variables by gender within each age group.

Age Group		Gender				Test Statistics	*p*
		Male		Female			
		Mean ± SD	Median (Min–Max)	Mean ± SD	Median (Min–Max)		
6–7 years	True Positive	33.29 ± 4.21	34 (26–39)	34.24 ± 4.54	35 (22–41)	181.5	0.425 *
	False Positive	1 ± 0.94	1 (0–3)	1 ± 1.47	0 (0–5)	184.5	0.444 *
	False Negative	1.29 ± 0.85	1 (0–3)	0.52 ± 0.71	0 (0–2)	107	0.004 *
	True Negative	0.53 ± 0.62	0 (0–2)	0.44 ± 0.65	0 (0–2)	192.5	0.555 *
	Errors	0.59 ± 0.71	0 (0–2)	1 ± 1.08	1 (0–3)	172.5	0.270 *
	Sensitivity	0.96 ± 0.02	0.97 (0.92–1)	0.98 ± 0.02	1 (0.94–1)	110	0.006 *
	Precision	0.97 ± 0.03	0.97 (0.9–1)	0.97 ± 0.05	1 (0.83–1)	189	0.527 *
	F1 Score	0.97 ± 0.02	0.97 (0.91–1)	0.98 ± 0.03	0.99 (0.89–1)	140	0.059 *
	False Discovery Rate	0.03 ± 0.03	0.03 (0–0.1)	0.03 ± 0.05	0 (0–0.17)	189	0.527 *
	False Negative Rate	0.04 ± 0.02	0.03 (0–0.08)	0.02 ± 0.02	0 (0–0.06)	110	0.006 *
8–9 years	True Positive	32.57 ± 3.69	33 (24–38)	30.58 ± 3.02	30 (25–37)	140	0.046 *
	False Positive	0.87 ± 1.18	0 (0–4)	1.58 ± 1.3	1 (0–4)	138.5	0.034 *
	False Negative	1 ± 1.54	0 (0–6)	0.63 ± 0.68	1 (0–2)	212	0.858 *
	True Negative	0.43 ± 0.66	0 (0–2)	0.74 ± 0.87	1 (0–3)	176.5	0.230 *
	Errors	1.35 ± 1.3	1 (0–4)	1.16 ± 1.26	1 (0–4)	199.5	0.618 *
	Sensitivity	0.97 ± 0.05	1 (0.83–1)	0.98 ± 0.02	0.97 (0.94–1)	218.5	1.000 *
	Precision	0.97 ± 0.04	1 (0.86–1)	0.95 ± 0.04	0.97 (0.86–1)	137.5	0.036 *
	F1 Score	0.97 ± 0.04	0.99 (0.86–1)	0.96 ± 0.02	0.97 (0.91–0.99)	139.5	0.045 *
	False Discovery Rate	0.03 ± 0.04	0 (0–0.14)	0.05 ± 0.04	0.03 (0–0.14)	137.5	0.036 *
	False Negative Rate	0.03 ± 0.05	0 (0–0.17)	0.02 ± 0.02	0.03 (0–0.06)	218.5	1.000 *
10 and more years	True Positive	27.25 ± 6.77	25 (20–43)	24.75 ± 1.89	25.5 (22–26)	22.5	0.854 *
	False Positive	1.58 ± 1.62	1.5 (0–4)	1.5 ± 1.29	1.5 (0–3)	24	1.000 *
	False Negative	0.67 ± 0.89	0 (0–2)	0.75 ± 0.96	0.5 (0–2)	22.5	0.839 *
	True Negative	0.33 ± 0.49	0 (0–1)	0.75 ± 0.96	0.5 (0–2)	18	0.394 *
	Errors	1 ± 0.74	1 (0–2)	0.75 ± 0.5	1 (0–1)	19.5	0.543 *
	Sensitivity	0.98 ± 0.03	1 (0.93–1)	0.97 ± 0.04	0.98 (0.92–1)	22	0.789 *
	Precision	0.94 ± 0.06	0.95 (0.85–1)	0.95 ± 0.04	0.94 (0.9–1)	24	1.000 *
	F1 Score	0.96 ± 0.03	0.96 (0.91–1)	0.96 ± 0.02	0.96 (0.93–0.98)	0.166	0.870 **
	False Discovery Rate	0.06 ± 0.06	0.05 (0–0.15)	0.05 ± 0.04	0.06 (0–0.1)	24	1.000 *
	False Negative Rate	0.02 ± 0.03	0 (0–0.07)	0.03 ± 0.04	0.02 (0–0.08)	22	0.789 *

* Mann–Whitney U test; ** independent samples *t*-test; mean ± standard deviation; median (minimum–maximum).

**Table 5:** Comparison of variables across age groups within each gender.

Gender		Age Group			Test Statistics	*p*
		6–7 Years	8–9 Years	10 and More Years		
Male	True Positive	33.29 ± 4.21	32.57 ± 3.69	27.25 ± 6.77	9.868	0.007 *
		34 (26–39) ^a^	33 (24–38) ^a^	25 (20–43) ^b^		
	False Positive	1 ± 0.94	0.87 ± 1.18	1.58 ± 1.62	1.795	0.408 *
		1 (0–3)	0 (0–4)	1.5 (0–4)		
	False Negative	1.29 ± 0.85	1 ± 1.54	0.67 ± 0.89	4.516	0.105 *
		1 (0–3)	0 (0–6)	0 (0–2)		
		36 (30–42)	35 (28–39)	28 (20–45)		
	True Negative	0.53 ± 0.62	0.43 ± 0.66	0.33 ± 0.49	0.744	0.689 *
		0 (0–2)	0 (0–2)	0 (0–1)		
	Errors	0.59 ± 0.71	1.35 ± 1.3	1 ± 0.74	4.067	0.131 *
		0 (0–2)	1 (0–4)	1 (0–2)		
	Sensitivity	0.96 ± 0.02	0.97 ± 0.05	0.98 ± 0.03	3.805	0.149 *
		0.97 (0.92–1)	1 (0.83–1)	1 (0.93–1)		
		0 (0–0.07)	0 (0–0.07)	0 (0–0.04)		
	Precision	0.97 ± 0.03	0.97 ± 0.04	0.94 ± 0.06	1.853	0.396 *
		0.97 (0.9–1)	1 (0.86–1)	0.95 (0.85–1)		
	F1 Score	0.97 ± 0.02	0.97 ± 0.04	0.96 ± 0.03	2.287	0.319 *
		0.97 (0.91–1)	0.99 (0.86–1)	0.96 (0.91–1)		
	False Discovery Rate	0.03 ± 0.03	0.03 ± 0.04	0.06 ± 0.06	1.853	0.396 *
		0.03 (0–0.1)	0 (0–0.14)	0.05 (0–0.15)		
	False Negative Rate	0.04 ± 0.02	0.03 ± 0.05	0.02 ± 0.03	3.805	0.149 *
		0.03 (0–0.08)	0 (0–0.17)	0 (0–0.07)		
Female	True Positive	34.24 ± 4.54	30.58 ± 3.02	24.75 ± 1.89	17.548	<0.001 *
		35 (22–41) ^b^	30 (25–37) ^a^	25.5 (22–26) ^a^		
	False Positive	1 ± 1.47	1.58 ± 1.3	1.5 ± 1.29	3.798	0.150 *
		0 (0–5)	1 (0–4)	1.5 (0–3)		
	False Negative	0.52 ± 0.71	0.63 ± 0.68	0.75 ± 0.96	0.566	0.753 *
		0 (0–2)	1 (0–2)	0.5 (0–2)		
		36 (25–43)	33 (29–39)	27 (24–30)		
	True Negative	0.44 ± 0.65	0.74 ± 0.87	0.75 ± 0.96	1.543	0.462 *
		0 (0–2)	1 (0–3)	0.5 (0–2)		
	Errors	1 ± 1.08	1.16 ± 1.26	0.75 ± 0.5	0.160	0.923 *
		1 (0–3)	1 (0–4)	1 (0–1)		
	Sensitivity	0.98 ± 0.02	0.98 ± 0.02	0.97 ± 0.04	0.943	0.624 *
		1 (0.94–1)	0.97 (0.94–1)	0.98 (0.92–1)		
		0 (0–0.06)	0.03 (0–0.12)	0.02 (0–0.08)		
	Precision	0.97 ± 0.05	0.95 ± 0.04	0.95 ± 0.04	4.822	0.090 *
		1 (0.83–1)	0.97 (0.86–1)	0.94 (0.9–1)		
	F1 Score	0.98 ± 0.03	0.96 ± 0.02	0.96 ± 0.02	7.435	0.024 *
		0.99 (0.89–1)	0.97 (0.91–0.99)	0.96 (0.93–0.98)		
	False Discovery Rate	0.03 ± 0.05	0.05 ± 0.04	0.05 ± 0.04	4.822	0.090 *
		0 (0–0.17)	0.03 (0–0.14)	0.06 (0–0.1)		
	False Negative Rate	0.02 ± 0.02	0.02 ± 0.02	0.03 ± 0.04	0.943	0.624 *
		0 (0–0.06)	0.03 (0–0.06)	0.02 (0–0.08)		

* Kruskal–Wallis test; mean ± standard deviation; median (minimum–maximum) ^a,b^: groups with the same letter have no statistically significant difference.

## 4. Discussion

This study evaluated the performance of an artificial intelligence-based system in detecting and numbering teeth in patients with cleft lip and palate. Overall, the AI system demonstrated high sensitivity (0.98 ± 0.03) and precision (0.96 ± 0.04), confirming its reliability in tooth detection across a wide patient cohort. Based on the findings of this study, the null hypothesis was rejected. While the commercially available AI system, Diagnocat, demonstrated overall high performance comparable to expert ground truth annotations, specific challenges were identified, particularly in the maxillary left region. These findings indicate that the AI-based software requires further refinement to address limitations in the cleft region.

Several studies have evaluated the use of AI systems for tooth detection and numbering, particularly focusing on panoramic radiographs and CBCTs [20,21,22]. In many of these studies, AI systems have demonstrated high sensitivity and precision, generally ranging from 0.90 to 0.95 [23]. These findings align with a growing body of research highlighting the potential of AI in various dental diagnostic tasks, including tooth identification and lesion detection [23]. The accuracy and consistency of the AI system presented here suggest that it can be a useful tool in assisting clinicians with dental numbering and detection, even in patients with complex conditions such as cleft lip and palate.

Moreover, the AI system’s performance across different age groups was also evaluated, which is crucial given the developmental changes in dentition, particularly in patients with cleft lip and palate. Analyzing mixed dentition presents unique challenges for clinicians due to the rapid developmental changes and variations in tooth eruption patterns [24]. One of the significant findings of this study is the lack of statistically significant differences in the AI system’s performance across different age groups. This, consistent with the existing literature, suggests that the AI model can effectively detect and number teeth in patients of varying ages, despite the rapid developmental changes that occur during the mixed dentition period [25]. Notably, in younger age groups, where dental development is still ongoing, the AI system performed with similar accuracy as in older age groups, such as those aged 10 and above. These findings highlight the potential applicability of AI systems in managing mixed dentition cases, where manual tooth detection can be more challenging due to the transitory nature of the dental arch.

Additionally, gender-based differences were identified, particularly in the younger age groups. Female participants in the 6–7 age group showed significantly higher sensitivity and lower false negative rates compared to their male counterparts. This suggests that the AI system may be more efficient in detecting teeth in female patients within this age range. In contrast, the 8–9 age group showed higher precision values for male participants, although the clinical significance of this remains unclear. These findings point to the possibility that anatomical or developmental factors, such as tooth eruption patterns or growth differences, may influence the performance of AI systems in different genders and age groups [26,27]. Further investigation is needed to better understand these gender-based disparities and their implications for the clinical application of AI-based dental imaging technologies.

The AI system’s consistent performance across different age groups aligns with the findings of Kim et al. [25], who also reported successful AI performance across varying patient ages. These studies suggest that age-related factors do not significantly affect AI performance. Our results further reinforce the idea that the AI model is robust in detecting and numbering teeth across a wide range of ages.

While the AI system demonstrated high overall performance in tooth detection and numbering, there are potential limitations that warrant further consideration. The study findings suggest that the AI model may face challenges in accurately identifying teeth in the maxillary left region, which exhibited higher false positive and false negative rates across all age groups. This may be attributed to the higher prevalence of cleft anomalies on the left side, as reported in the literature [28]. Among the individuals included in this study, unilateral left clefts were more common, consistent with previous research indicating that cleft lip and palate cases are predominantly unilateral and more frequently affect the left side [29]. Specifically, tooth number 21 had the highest false positive rate (16%), while tooth number 62 had the highest false negative rate (11%). These teeth are located in the regions most affected by cleft anomalies, particularly in patients with left-sided clefts, further emphasizing the challenges AI faces in accurately detecting and numbering teeth in these areas.

Additionally, the observed gender-based differences, particularly in the younger age groups, suggest that the AI system may not be equally effective in detecting teeth across all patient populations. These findings indicate the need for the further refinement and customization of AI algorithms to better address the unique dental characteristics and developmental variations seen in cleft lip and palate patients. While the overall performance of the AI system is promising, the limitations highlighted in this study underscore the importance of continued research and validation to ensure the technology can be applied effectively and equitably across diverse patient demographics [30,31].

The AI system’s consistent performance across different age and gender groups observed in this study may reflect the robustness of Diagnocat’s training process. As a commercially available tool, Diagnocat has been trained by its developers using diverse datasets to optimize its accuracy and generalizability. However, since the specifics of the training data, including demographic distributions, are proprietary, further studies could explore how variations in training datasets might influence AI performance in populations with unique anatomical challenges, such as cleft lip and palate patients.

## 5. Conclusions

This study demonstrates the strong potential of artificial intelligence (AI) to detect and number teeth in patients with cleft lip and palate, with the AI system showing high sensitivity and precision overall. The system performed well across various age groups, proving its robustness in managing the unique dental challenges that these patients present. Future improvements focused on the cleft area could further elevate its clinical utility, leading to better diagnosis and management in this population.

## Figures and Tables

**Table 1:** The descriptive statistics for the variables studied across all patients included in the study.

	Mean ± SD	Median (Min–Max)
True Positive	31.78 ± 4.96	33 (20–43)
False Positive	1.17 ± 1.31	1 (0–5)
False Negative	0,81 ± 1.02	1 (0–6)
True Negative	0.51 ± 0.69	0 (0–3)
Errors	1.03 ± 1.08	1 (0–4)
Sensitivity	0.98 ± 0.03	0.97 (0.83–1)
Precision	0.96 ± 0.04	0.97 (0.83–1)
F1 Score	0.97 ± 0.03	0.97 (0.86–1)
False Discovery Rate	0.04 ± 0.04	0.03 (0–0.17)
False Negative Rate	0.02 ± 0.03	0.03 (0–0.17)

SD, Standard Deviation.

**Table 2:** Comparison of the AI system’s sensitivity, precision, and specificity across the three age groups.

	Age Group			Test Statistics	*p* *
	6–7 Years	8–9 Years	10 and More Years		
True Positive	33.86 ± 4.38	31.67 ± 3.51	26.63 ± 5.97	23.810	<0.001 *
	35 (22–41) ^c^	32 (24–38) ^b^	25 (20–43) ^a^		
False Positive	1 ± 1.27	1.19 ± 1.27	1.56 ± 1.5	1.859	0.395
	1 (0–5)	1 (0–4)	1.5 (0–4)		
False Negative	0.83 ± 0.85	0.83 ± 1.23	0.69 ± 0.87	0.660	0.719
	1 (0–3)	0.5 (0–6)	0 (0–2)		
	36 (25–43) ^c^	33 (28–39) ^b^	27 (20–45) ^a^		
True Negative	0.48 ± 0.63	0.57 ± 0.77	0.44 ± 0.63	0.293	0.864
	0 (0–2)	0 (0–3)	0 (0–2)		
Errors	0.83 ± 0.96	1.26 ± 1.27	0.94 ± 0.68	2.354	0.308
	1 (0–3)	1 (0–4)	1 (0–2)		
Sensitivity	0.98 ± 0.02	0.97 ± 0.04	0.98 ± 0.03	0.349	0.840
	0.97 (0.92–1)	0.99 (0.83–1)	1 (0.92–1)		
	0 (0–0.07)	0 (0–0.12)	0 (0–0.08)		
Precision	0.97 ± 0.04	0.96 ± 0.04	0.94 ± 0.05	3.521	0.172
	0.97 (0.83–1)	0.97 (0.86–1)	0.94 (0.85–1)		
F1 Score	0.97 ± 0.03	0.97 ± 0.03	0.96 ± 0.03	2.727	0.256
	0.97 (0.89–1)	0.98 (0.86–1)	0.96 (0.91–1)		
False Discovery Rate	0.03 ± 0.04	0.04 ± 0.04	0.06 ± 0.05	3.521	0.172
	0.03 (0–0.17)	0.03 (0–0.14)	0.06 (0–0.15)		
False Negative Rate	0.02 ± 0.02	0.03 ± 0.04	0.02 ± 0.03	0.349	0.840
	0.03 (0–0.08)	0.01 (0–0.17)	0 (0–0.08)		

* *p* = Kruskal–Wallis test; mean ± standard deviation; median (minimum–maximum) ^a–c^: groups with the same letter have no statistically significant difference.

**Table 3:** Gender comparisons for sensitivity, precision, and F1 score.

	Gender				Test Statistics	*p* *
	Male		Female			
	Mean ± SD	Median (Min–Max)	Mean ± SD	Median (Min–Max)		
True Positive	31.58 ± 5.21	33 (20–43)	32 ± 4.71	33 (22–41)	1195.5	0.716
False Positive	1.08 ± 1.23	1 (0–4)	1.27 ± 1.4	1 (0–5)	1155.5	0.503
False Negative	1.02 ± 1.21	1 (0–6)	0.58 ± 0.71	0 (0–2)	1013	0.079
True Negative	0.44 ± 0.61	0 (0–2)	0.58 ± 0.77	0 (0–3)	1152	0.448
Errors	1.02 ± 1.06	1 (0–4)	1.04 ± 1.11	1 (0–4)	1246	0.988
Sensitivity	0.97 ± 0.04	0.97 (0.83–1)	0.98 ± 0.02	1 (0.92–1)	1035	0.119
Precision	0.96 ± 0.04	0.97 (0.85–1)	0.96 ± 0.05	0.97 (0.83–1)	1140	0.441
F1 Score	0.97 ± 0.03	0.97 (0.86–1)	0.97 ± 0.03	0.98 (0.89–1)	1216.5	0.827
False Discovery Rate	0.04 ± 0.04	0.03 (0–0.15)	0.04 ± 0.05	0.03 (0–0.17)	1140	0.441
False Negative Rate	0.03 ± 0.04	0.03 (0–0.17)	0.02 ± 0.02	0 (0–0.08)	1035	0.119

* *p* = Mann–Whitney U test; mean ± standard deviation; median (minimum–maximum); *p* > 0.05.

## Data Availability

The data presented in this study are available on request from the corresponding author.
